# Comparison of Leadless Pacing and Temporary Externalized Pacing Following Cardiac Implanted Device Extraction

**DOI:** 10.19102/icrm.2019.101204

**Published:** 2019-12-15

**Authors:** Holly Gonzales, Travis D. Richardson, Jay A. Montgomery, George H. Crossley, Christopher R. Ellis

**Affiliations:** ^1^Cardiovascular Division, Vanderbilt Heart and Vascular Institute, Vanderbilt University Medical Center, Nashville, TN, USA

**Keywords:** Device infection, leadless pacing, pacemaker, pacemaker-dependent, transvenous lead extraction

## Abstract

Pacemaker-dependent (PD) patients undergoing implantable cardiac electronic device extraction often must be subjected to temporary pacing interventions. We sought to determine the safety and utility of a leadless pacing system (Micra™; Medtronic, Minneapolis, MN, USA) in patients undergoing system extraction as compared with externalized temporary transvenous right ventricular lead (temp-perm) placement. We performed a retrospective cohort analysis of all patients receiving either permanent Micra™ or temp-perm systems following system extraction from October 2013 to September 2017 at Vanderbilt University Hospital. The Micra™ and temp-perm cohorts included nine and 27 patients meeting the inclusion criteria, respectively. System infection was the most common indication for extraction (67% Micra™, 84% temp-perm), but no patients had active bacteremia at the time of permanent system reimplantation. There was no difference in system type (p = 0.09) or mean lead dwell time extracted (109 versus 81 months; p = 0.93). Procedure times were comparable between the two groups (180 versus 194 minutes; p = 0.74). Patients receiving Micra™ systems had shorter hospital stays after extraction (two versus eight days; p < 0.005), with no difference in major complications (11% versus 15%; p = 0.78) or 30-day (11% versus 7%; p = 0.77) or 90-day (11% versus 11%; p = 0.45) mortality. No reinfections were observed in either group at 90 days. Implantation of the Micra™ pacing system in select PD patients after system extraction is feasible and appears to reduce the hospital length of stay as compared with the use of temp-perm systems.

## Introduction

Cardiac implantable electronic device (CIED) implantation has been increasing since 2004 following trials demonstrating improved morbidity and mortality of implantable cardioverter-defibrillators (ICDs) and cardiac resynchronization therapy (CRT) devices.^1^ Due to an increase in implants, patient medical complexity, and population age, system extractions are also increasing.^2^ Device infection requiring system removal has been reported in 1.6% to 2.2% of implants, making CIED infection the leading indication for transvenous lead extraction, carrying a one-year mortality rate of up to 20%.^3–5^ Outcomes are improved with early and complete device extraction in the setting of pocket- or system-related infection as is reflected in current guidelines.^6^ Less common indications for extraction include lead malfunction, chronic pain, thrombosis, and symptomatic venous occlusion.

When CIED-related endocarditis is suspected, the typical practice is to provide 14 days to six weeks or more of intravenous antibiotics after extraction and prior to reimplantation in the contralateral side.^1^ Earlier reimplantation (up to 72 hours after extraction) is becoming more common for isolated pocket infection; however, extraction in pacemaker-dependent (PD) patients is more complex and costly due to the need for continuous pacing support. Passive temporary pacing wires may be less stable; as such, their use may require patient immobility, specialized monitoring, and prolonged inpatient hospitalization. Methods for more secure temporary pacing have been adapted, such as the insertion of active fixation leads through the internal jugular vein attached to a sterilized externalized pulse generator (PG) at the base of the neck.^7,8^

The Micra™ transcatheter pacing system (Medtronic, Minneapolis, MN, USA) is a 2.6-cm × 0.7-cm device with a Parylene polymer coating and titanium and nitinol tines, delivered through a 23-French (Fr) delivery system via the femoral vein.^9^ The device was the first leadless pacemaker approved by the United States Food and Drug Administration for use.^10^ In the Micra™ investigational study, the implantation success rate was high, thresholds were low and stable at six months, and major complications were reduced relative to those in a transvenous comparison group, with no device infections observed for up to six months after implantation.^11,12^ Since there is no device pocket and minimal intravascular foreign material introduced during placement, the use of the Micra™ system following CIED extraction (assuming the resolution of any active infections) seems feasible and may minimize future device infection. Further, this approach could obviate the need for a waiting period with other forms of temporary pacing support in PD patients following extraction, thus decreasing the duration of hospitalization. We sought to establish the feasibility of using this device at the time of CIED extraction as an alternative to the active fixation lead with an externalized PG approach in PD patients undergoing extraction.

## Materials and methods

### Study cohorts

All patients receiving either externalized active fixation leads with sterilized PGs (temp-perm cohort) or Micra™ devices (Micra™ cohort) after CIED system extraction at Vanderbilt University Medical Center between October 1, 2013, and September 1, 2017 were included in this study for retrospective analysis. This study was approved by the institutional review board at Vanderbilt University Medical Center. Medical records were reviewed for patient demographic information, CIED infection risk factors, and procedural and outcome variables.^13^ The decision to pursue use of a temp-perm or a Micra™ system, respectively, was made by the operator. However, our current institutional practice mandates the use of the temp-perm approach in patients with ongoing bacteremia.

### Extraction technique

All procedures were performed in a hybrid electrophysiology laboratory/operating room by an electrophysiologist experienced in transvenous lead extraction with immediate cardiac surgery backup available. Relevant supplies were available, including blood products, a rapid infuser, sternal saw, Cell Saver^®^ (Haemonetics Corp., Braintree, MA, USA), and cardiac surgery operating room trays. Powered sheaths (12–16-Fr Spectranetics GlideLight SLS; Spectranetics Corp., Colorado Springs, CO, USA) as well as mechanical extraction tools (Spectranetics TightRail™ and Visi Sheath; Spectranetics Corp., Colorado Springs, CO, USA) and femoral snares (15–25-mm ONE Snare^®^ Gooseneck Snare; Merit Medical Systems, Malvern, PA, USA) were used as necessary to achieve complete system extraction. Continuous central venous access and arterial pressure monitoring were maintained throughout the procedure. Temporary pacing wires during the index extraction were placed via a femoral approach in the PD patients. Implantation of either study device was performed immediately following system extraction, during the same procedure.

### Temp-perm

**Figure 1:**
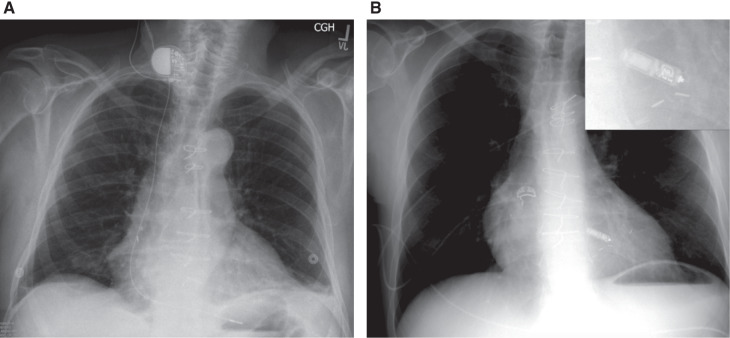
Implanted devices. **A:** Chest radiograph demonstrating active fixation lead introduced through the right internal jugular vein with an attachment to an externalized PG. **B:** Chest radiograph demonstrating positioning of the Micra™ device (Medtronic, Minneapolis, MN, USA) (magnified inset).

Temp-perm devices were placed via percutaneous access to either the internal jugular or axillary veins. Active fixation model 5076 leads (Medtronic, Minneapolis, MN, USA) were placed through a 7-Fr peel-away sheath advanced to the right ventricular (RV) apex or septum according to the operator’s discretion. The lead was sutured to the skin at the suture collar and the entry site was covered with a sterile dressing. The lead was then attached to a reprocessed sterilized or new PG (Adapta or Sensia models; Medtronic, Minneapolis, MN, USA) and anchored to the adjacent skin with a sterile dressing **(Figure 1A)**.

### Micra™

The Micra™ leadless pacemaker system (LPS) was inserted via its 23-Fr access sheath in the right femoral vein using a previously described technique.^9,11^ Venous access was initially obtained with a 9-Fr short sheath, through which 20 mL of contrast was administered to ensure suitable anatomy. A 180-cm Amplatzer Super Stiff guidewire (Boston Scientific, Natick, MA, USA) was then advanced into the superior vena cava and the access site was subsequently dilated to a 16-Fr size with insertion of the aforementioned 23-Fr access sheath, which was advanced to the midpoint of the right atrium. A Micra™ delivery catheter (Medtronic, Minneapolis, MN, USA) was used to position the device in the RV apex or septum according to the operator’s discretion **(Figure 1B)**. Tine stability evaluations and tug testing were performed, followed by the completion of electrical testing prior to device release. Following good results, the access site was closed with manual hemostasis and a figure-of-eight stitch, followed by two hours of bed rest.

### Follow-up

Procedural data such as procedure duration, extraction system details, major complications, and hospital length of stay (LOS) were collected from the medical record of the index hospitalization for CIED extraction. Mortality at 30 days and 90 days was assessed by chart review. Procedure times include the index procedure time for both groups (initial extraction with either temp-perm or Micra™ implantation) and total procedure time (extraction with temp-perm implantation added to the eventual permanent device reimplantation procedure time). Average lead dwell time was defined as the mean age in months of all previously implanted leads divided by the number of leads present. Hospital duration was defined as the time from index extraction to hospital discharge, with in-hospital mortalities excluded. Major complications were defined according to the 2017 Heart Rhythm Society Consensus statement on transvenous lead extraction as “those that pose an immediate threat to life or that result in death.” Examples include death, cardiac avulsion requiring procedural intervention, vascular injury requiring intervention, and pericardial effusion requiring intervention.^6^

### Statistical analysis

Patient characteristics are expressed as means and standard deviations. Univariate comparisons of patient characteristics, procedural characteristics, and outcomes were performed using chi-squared tests for categorical variables or the Student’s t-test for continuous variables. A two-tailed p-value of 0.05 or less was considered to be statistically significant. Statistical analyses were performed using Microsoft Excel version 15.22 (Microsoft Corp., Redmond, WA, USA).

## Results

During the study period, 27 patients underwent implantation of a temp-perm pacing system and nine patients underwent implantation of the Micra™ LPS at the time of extraction. There were no statistically significant differences in terms of age, gender, extraction indication, type of CIED extracted, anticoagulation use, or average lead dwell time (defined as the total lead age divided by the number of extracted leads) between the two groups **(Table 1)**. Many patients underwent extraction of a defibrillator (59% in the temp-perm cohort and 22% in the Micra™ cohort; p = 0.09). As anticipated, due to the perceived need for either CRT or transvenous ICD therapy, no patients in the Micra™ cohort had an ejection fraction of less than 35% as compared with 15% of the patients in the temp-perm cohort (p = 0.04). The average lead dwell time in both groups was more than six years (p = 0.93), with the temp-perm group having a slightly higher number of explanted leads per procedure (three versus two leads; p = 0.009). Despite this, the index procedure time was similar in both cohorts (180 minutes for the Micra™ cohort versus 194 minutes for the temp-perm cohort; p = 0.74). When total procedure times were calculated by adding in the time for reimplantion of the temp-perm cohort’s replacement CIEDs, Micra™ procedure times were significantly less (180 minutes versus 325 minutes; p < 0.001). Further, the duration of hospitalization following device extraction among patients discharged from the hospital was decreased by an average of six days in the Micra™ cohort (eight versus two days; p < 0.001).

System infection was the most common indication for extraction (67% Micra™ versus 84% temp-perm). All patients in the Micra™ cohort had negative blood cultures prior to implantation, and these patients were less likely to have fever or leukocytosis in the 24 hours prior to implantation (11% versus 37% in the temp-perm cohort; p = 0.04). Other risk factors for device infection between cohorts were similar. We observed no differences in major complication rates or 30-day or 90-day mortality between cohorts **(Table 2)**. Additionally, no device reinfections were observed in either group.

Regarding mortality rates, there were three deaths in the temp-perm group, with two occurring during the index hospitalization for extraction as a result of vascular injury requiring surgical repair followed by intractable shock and procedural hemopericardium with tamponade. The third death occurred 38 days after extraction out of the hospital from an unknown cause; in this case, the temp-perm system had been utilized for four weeks of intravenous antibiotics until implantation of a new transvenous system. Separately, there was one death in the Micra™ cohort that occurred during the index hospitalization period for lead extraction due to severe lead-related tricuspid regurgitation. After hybrid tricuspid valve replacement and extraction, the patient developed ischemic bowel and died on the 28^th^ day in the hospital.

## Discussion

Observation of CIED-related complications such as pocket and lead infections, lead fractures and recalls, and vascular complications have led to the long-awaited advent of leadless pacemaker and defibrillator systems. The Micra™ system has many potential advantages over a transvenous system with externalized PG, many of which are particularly beneficial in patients undergoing lead extraction. Namely, an LPS appears to confer a lower risk of device infection and avoids the need for pocket formation/manipulation and upper-extremity venous access.^14^ A recent case series reported on 17 patients who underwent LPS implantation both early (less than one week) and late (more than one week) after system extraction for infection and observed no LPS-related infections. In this series, PD patients used a temp-perm system to bridge to eventual LPS implantation.^15^ Our series demonstrates that, in highly selected patients with indications for single-chamber pacing without evidence of ongoing bacteremia, a Micra™ system can be considered as an alternative to an externalized active fixation pacing system after lead extraction when continuous pacing support is required.

Micra™ implantation after extraction at our center did not take significantly longer than that in the context of a temp-perm system. Additionally, when reimplantation time was considered for the temp-perm cohort, the combined procedure times were reduced. Longer procedure times associated with temp-perm use could theoretically correlate with increased morbidity in the form of longer fluoroscopy, anesthesia time, and increased infection risk.

**Figure 2:**
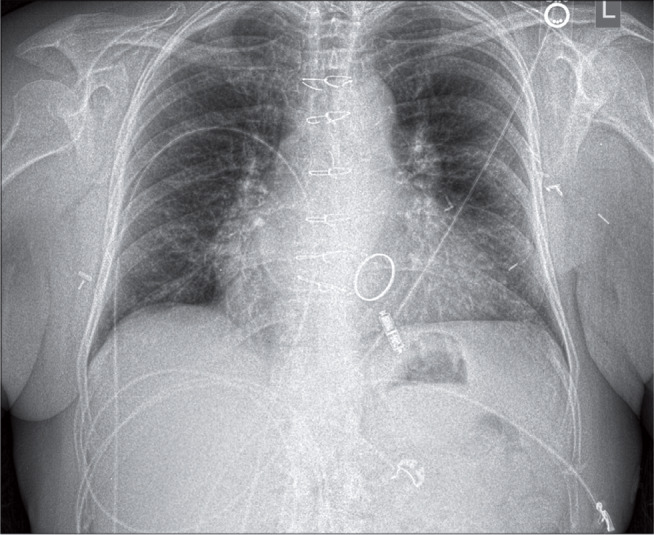
Illustrative case. Chest radiograph demonstrating Micra™ device (Medtronic, Minneapolis, MN, USA) placement in RV apical position after extraction of single-chamber pacemaker in a patient with a mechanical mitral valve.

The most striking observation in the Micra™ cohort was the significant reduction in hospital LOS when compared with the temp-perm approach. In addition to this group’s decreased risk attributed to higher left ventricular ejection fraction and cleared bacteremia prior to implantation, this reduction can also be explained by the lack of a waiting period after CIED extraction prior to system reimplant in the Micra™ group. Patients in the temp-perm group mostly underwent extraction for system infection and, as such, were treated with antibiotics for some period prior to implantation of a permanent CIED system. Between 2013 and 2017, the majority of patients were required to be admitted with their externalized PG system. In contrast, patients in the LPS group with infection underwent Micra™ implantation during the same procedure immediately following system extraction **(Figure 2)**.

Acknowledging the differences in the patient populations compared, we observed no difference in mortality or major complications between patients undergoing Micra™ implantation following CIED extraction as compared with use of an externalized active fixation pacing system. In our study, patients were only eligible for the former if they did not have active bacteremia at the time of CIED system extraction or other evidence of ongoing infection. We felt this was a safe alternative to temp-perm placement in patients without active bacteremia given the small profile of the Micra™ system in the bloodstream, its small surface area, and the rapid endothelialization of this kind of device.^16^ In fact, a large retrospective study of all registered Micra™ revisions suggested no device-related infections prompting extraction have occurred.^17^ The strategy of implanting an LPS at the time of extraction was previously demonstrated to be safe in a small single-center case series and larger retrospective cohort analysis.^18,19^ Despite the implantation of an intravascular device immediately following laser lead extraction, we did not observe any cases of Micra™-associated infection in this group.

It is important to note that there are limitations to the use of a Micra™ device. Currently, the LPS can only provide VVI(R) pacing support, making it a less attractive option for patients with atrioventricular (AV) block in sinus rhythm, sinus node dysfunction with high pacing burdens, or patients requiring CRT pacing. However, investigators in the United Kingdom Pacing and Cardiovascular Events (UKPACE) trial showed that elderly patients with high-grade AV block may not benefit from dual-chamber pacing.^20^ Given our study’s median age of 70 years, even patients with AV block could represent a group that might benefit from single-chamber pacing with an LPS, notwithstanding well-known complications with high percentages of RV pacing including increased rates of heart failure and atrial fibrillation. Indeed, four of our patients underwent Micra™ implantation in the setting of sinus rhythm and AV block, with none requiring system revision for pacemaker syndrome. Our center has previously revised other patients initially receiving these devices to transvenous systems without difficulty by adding transvenous leads and reprogramming their Micra™ system to ODO. If needed, Micra™ retrieval has been shown to be safe and successful within up to 14 months after implantation.^17^

Further, the Micra™ system has no defibrillator capabilities. Because of this, patients needing ICD therapy require an additional subcutaneous ICD implantation or implantation of another transvenous ICD system as previously described.^16^ The Micra™ system also has a higher cost than a traditional transvenous lead system; however, it is possible that the significant reduction in the hospital LOS observed in the LPS cohort may still support Micra™ as a cost-effective approach as compared with the use of externalized PGs. Further research to address this question should be pursued.

There may ultimately be patient populations in whom Micra™ implantation is preferred over the use of transvenous systems, although no randomized data supporting such currently exists. Examples could include permanent atrial fibrillation with bradycardia, end-stage renal disease with limited vascular access, previous bilateral pectoral pocket infections, poor wound healing, and a lack of pectoral soft tissue as is typically seen in frail or elderly adults.

### Study limitations

This was a small, single-center, retrospective study with a degree of selection bias inherent to this type of observational study, notably creating differences between the two cohorts with respect to global cardiovascular risk. Further, the small sample size limits our power to detect minute differences in mortality and complication rates that may be observed between groups, although we saw no suggestion of a trend of increased risk in the Micra™ cohort. Furthermore, due to the small sample size of our study, a multivariate analysis was not appropriate. Lastly, our follow-up period was relatively short, limiting our ability to detect longer-term differences in mortality, reinfection rate (given that reinfection could take up to a year to manifest), or the need for reintervention with a transvenous system after Micra™ implantation.

## Conclusion

The use of an LPS in carefully selected PD patients undergoing lead extraction is feasible for select patients. This strategy may reduce the hospital LOS following extraction and does not appear to confer additional short-term mortality as compared with the use of an externalized transvenous pacing system prior to CIED reimplantation. Larger-scale prospective studies are needed to verify the long-term safety of such an approach.

## Figures and Tables

**Table 1:** Patient Baseline Characteristics

Characteristics	Cohorts		p-value
	Temp-perm (n = 27)	Micra™ (n = 9)	
Age, mean ± SD	69.7 ± 12.2 years	66.1 ± 15.1 years	0.456
Male gender, n (%)	20 (74%)	8 (78%)	0.415
Device extracted, n (%)			0.088
PPM	9 (33%)	6 (67%)	
CRT-P	2 (7%)	1 (11%)	
ICD	4 (15%)	0	
CRT-D	12 (44%)	2 (22%)	
No. of leads extracted, mean ± SD	3 ± 0.8	2 ± 0.9	0.009
Average lead dwell time, mean ± SD	81 ± 52.2 months	109.3 ± 83.5 months	0.934
Indication for extraction, n (%)			0.672
Pocket infection	14 (52%)	5 (56%)	
Device-related endocarditis	9 (33%)	1 (11%)	
Lead malfunction	2 (7%)	2 (22%)	
Valvular disease	2 (7%)	1 (11%)	
HFrEF (LVEF < 35%)	4 (15%)	0	0.043
CIED risk factors, n (%)			
Diabetes	11 (41%)	3 (33%)	0.353
CKD (Cr ; 1.5)	6 (22%)	0	0.216
Immunosuppressants	5 (19%)	2 (22%)	0.413
Active malignancy	1 (4%)	0	0.163
Structural HD	7 (26%)	2 (22%)	0.415
Recent pocket manipulation	10 (37%)	1 (11%)	0.045
Chronic CVC	4 (15%)	0	0.022
IVDU	1 (4%)	1 (11%)	0.271
Fever/leukocytosis	10 (37%)	1 (11%)	0.045
History of CIED infection	7 (26%)	3 (33%)	0.350
Chronic anticoagulation	16 (60%)	6 (67%)	0.353

CIED: cardiac implantable electronic device; CKD: chronic kidney disease; Cr: serum creatinine; CRT-D: cardiac resynchronization therapy defibrillator; CRT-P: cardiac resynchronization therapy pacemaker; CVC: central venous catheter; HFrEF: heart failure with reduced ejection fraction; HD: heart disease; ICD: implantable cardioverter defibrillator; IVDU: intravenous drug use; n: number; PPM: permanent pacemaker; SD: standard deviation.

**Table 2:** Measured Outcomes

Outcomes	Cohorts		p-value
	Temp-perm (n = 27)	Micra™ (n = 9)	
Index procedure duration, mean ± SD	193.7 ± 79.9 min	180 ± 45.2 min	0.738
Total procedure duration, mean ± SD	324.6 ± 92 min	180 ± 45.2 min	0.0002
Hospital length of stay, mean ± SD	8.5 ± 5.2 days	2.3 ± 2.1 days	0.00004
Major complications, n (%)	4 (15%)	1 (11%)	0.782
30-day mortality	7%	11%	0.768
90-day mortality	11%	11%	0.451

n: number; SD: standard deviation.
